# Supplementary material for: Transcriptome Analysis Reveals Vimentin-Induced Disruption of Cell–Cell Associations Augments Breast Cancer Cell Migration
Source: Cells. 2022 Dec 13;11(24):4035. doi: 10.3390/cells11244035 (PMC9776984; doi:10.3390/cells11244035)
Supplement: Supplementary file 1 [file cells-11-04035-s001.zip › DEGs and enrichment analysis.zip!/DEGs and enrichment analysis/WT_VIMvsVector GO enrichment/GSEA/WT_VIM/MUSCLE_FILAMENT_SLIDING/MUSCLE_FILAMENT_SLIDING(GO_0030049).html]

Details for gene set MUSCLE\_FILAMENT\_SLIDING(GO:0030049)[GSEA]


|  || Dataset | fpkm.sample |
| Phenotype | sample.cls |
| Upregulated in class | WT\_VIM |
| GeneSet | MUSCLE\_FILAMENT\_SLIDING(GO:0030049) |
| Enrichment Score (ES) | 0.93824327 |
| Normalized Enrichment Score (NES) | 1.4725091 |
| Nominal p-value | 0.0 |
| FDR q-value | 0.16299967 |
| FWER p-Value | 0.163 |
Table: GSEA Results Summary

  

Fig 1: Enrichment plot: MUSCLE\_FILAMENT\_SLIDING(GO:0030049)
  
Profile of the Running ES Score & Positions of GeneSet Members on the Rank Ordered List

  

| PROBE | DESCRIPTION   (from dataset) | GENE SYMBOL | GENE\_TITLE | RANK IN GENE LIST | RANK METRIC SCORE | RUNNING ES | CORE ENRICHMENT || 1 | ENSG00000086967 | MYBPC2 |  |  | 1906 | Infinity | -0.0322 | Yes |
| 2 | ENSG00000175084 | DES |  |  | 2208 | Infinity | -0.0373 | Yes |
| 3 | ENSG00000026025 | VIM |  |  | 2388 | 1709.539 | 0.9375 | Yes |
| 4 | ENSG00000248746 | ACTN3 |  |  | 2610 | 7.924 | 0.9382 | Yes |
| 5 | ENSG00000105048 | TNNT1 |  |  | 3454 | 3.697 | 0.9261 | No |
| 6 | ENSG00000129991 | TNNI3 |  |  | 3477 | 3.690 | 0.9278 | No |
| 7 | ENSG00000130598 | TNNI2 |  |  | 4008 | 3.012 | 0.9206 | No |
| 8 | ENSG00000196465 | MYL6B |  |  | 5582 | 2.166 | 0.8952 | No |
| 9 | ENSG00000077522 | ACTN2 |  |  | 5601 | 2.159 | 0.8961 | No |
| 10 | ENSG00000159173 | TNNI1 |  |  | 6898 | 1.841 | 0.8753 | No |
| 11 | ENSG00000101306 | MYLK2 |  |  | 8022 | 1.619 | 0.8572 | No |
| 12 | ENSG00000114854 | TNNC1 |  |  | 9359 | 1.435 | 0.8354 | No |
| 13 | ENSG00000173991 | TCAP |  |  | 10279 | 1.327 | 0.8206 | No |
| 14 | ENSG00000101470 | TNNC2 |  |  | 12226 | 1.162 | 0.7883 | No |
| 15 | ENSG00000143549 | TPM3 |  |  | 12404 | 1.148 | 0.7860 | No |
| 16 | ENSG00000092841 | MYL6 |  |  | 12827 | 1.116 | 0.7795 | No |
| 17 | ENSG00000183091 | NEB |  |  | 12996 | 1.102 | 0.7773 | No |
| 18 | ENSG00000198947 | DMD |  |  | 14471 | 0.996 | 0.7529 | No |
| 19 | ENSG00000136842 | TMOD1 |  |  | 16244 | 0.903 | 0.7234 | No |
| 20 | ENSG00000140416 | TPM1 |  |  | 20795 | 0.646 | 0.6468 | No |
| 21 | ENSG00000118194 | TNNT2 |  |  | 20959 | 0.637 | 0.6444 | No |
| 22 | ENSG00000155657 | TTN |  |  | 22061 | 0.573 | 0.6261 | No |
| 23 | ENSG00000143632 | ACTA1 |  |  | 22202 | 0.563 | 0.6241 | No |
| 24 | ENSG00000167460 | TPM4 |  |  | 24496 | 0.399 | 0.5855 | No |
| 25 | ENSG00000198467 | TPM2 |  |  | 25123 | 0.334 | 0.5751 | No |
| 26 | ENSG00000109063 | MYH3 |  |  | 26268 | 0.220 | 0.5559 | No |
| 27 | ENSG00000198336 | MYL4 |  |  | 27497 | 0.000 | 0.5351 | No |
| 28 | ENSG00000196091 | MYBPC1 |  |  | 29635 | 0.000 | 0.4989 | No |
| 29 | ENSG00000092054 | MYH7 |  |  | 30803 | 0.000 | 0.4792 | No |
| 30 | ENSG00000264424 | MYH4 |  |  | 37661 | NaN | 0.3632 | No |
| 31 | ENSG00000168530 | MYL1 |  |  | 40484 | NaN | 0.3154 | No |
| 32 | ENSG00000111245 | MYL2 |  |  | 45445 | NaN | 0.2315 | No |
| 33 | ENSG00000197616 | MYH6 |  |  | 51542 | NaN | 0.1283 | No |
| 34 | ENSG00000160808 | MYL3 |  |  | 51787 | NaN | 0.1242 | No |
| 35 | ENSG00000134571 | MYBPC3 |  |  | 51831 | NaN | 0.1235 | No |
| 36 | ENSG00000159251 | ACTC1 |  |  | 51907 | NaN | 0.1222 | No |
| 37 | ENSG00000125414 | MYH2 |  |  | 53864 | NaN | 0.0891 | No |
| 38 | ENSG00000133020 | MYH8 |  |  | 55163 | NaN | 0.0672 | No |
| 39 | ENSG00000130595 | TNNT3 |  |  | 58618 | NaN | 0.0087 | No |
Table: GSEA details
[plain text format]

  

Fig 2: MUSCLE\_FILAMENT\_SLIDING(GO:0030049)
  
Blue-Pink O' Gram in the Space of the Analyzed GeneSet

  

Fig 3: MUSCLE\_FILAMENT\_SLIDING(GO:0030049): Random ES distribution
  
Gene set null distribution of ES for
 **MUSCLE\_FILAMENT\_SLIDING(GO:0030049)**
